# Mapping each pre-existing condition’s association to short-term and long-term COVID-19 complications

**DOI:** 10.1038/s41746-021-00484-7

**Published:** 2021-07-27

**Authors:** A. J. Venkatakrishnan, Colin Pawlowski, David Zemmour, Travis Hughes, Akash Anand, Gabriela Berner, Nikhil Kayal, Arjun Puranik, Ian Conrad, Sairam Bade, Rakesh Barve, Purushottam Sinha, John C. O‘Horo, Andrew D. Badley, John Halamka, Venky Soundararajan

**Affiliations:** 1 nference, Cambridge, MA USA; 2 nference Labs, Bangalore, India; 3 Mayo Clinic, Rochester, MN USA

**Keywords:** Outcomes research, Predictive markers

## Abstract

Understanding the relationships between pre-existing conditions and complications of COVID-19 infection is critical to identifying which patients will develop severe disease. Here, we leverage ~1.1 million clinical notes from 1803 hospitalized COVID-19 patients and deep neural network models to characterize associations between 21 pre-existing conditions and the development of 20 complications (e.g. respiratory, cardiovascular, renal, and hematologic) of COVID-19 infection throughout the course of infection (i.e. 0–30 days, 31–60 days, and 61–90 days). Pleural effusion was the most frequent complication of early COVID-19 infection (89/1803 patients, 4.9%) followed by cardiac arrhythmia (45/1803 patients, 2.5%). Notably, hypertension was the most significant risk factor associated with 10 different complications including acute respiratory distress syndrome, cardiac arrhythmia, and anemia. The onset of new complications after 30 days is rare and most commonly involves pleural effusion (31–60 days: 11 patients, 61–90 days: 9 patients). Lastly, comparing the rates of complications with a propensity-matched COVID-negative hospitalized population confirmed the importance of hypertension as a risk factor for early-onset complications. Overall, the associations between pre-COVID conditions and COVID-associated complications presented here may form the basis for the development of risk assessment scores to guide clinical care pathways.

## Introduction

The COVID-19 pandemic remains an ongoing public health crisis^1^, and it is critically important to understand the full spectrum of complications that arise throughout the course of SARS-CoV-2 infection. There are already several emerging reports of risk factors of severe disease as well as lingering long-term effects such as fatigue, myalgia, and renal complications^2^. However, there is an incomplete understanding of the relationship between pre-existing comorbidities and post-COVID complications.

Automated curation of clinical notes affords the ability to rapidly perform epidemiologic studies from unstructured text found in electronic health records (EHRs). Previous efforts have leveraged various models for natural language processing to extract information regarding diagnoses, treatments, and clinical courses from unstructured data^3^. We have previously benchmarked different natural language processing models and transformer architectures^4^ and developed BERT-based models to curate unstructured clinical data from EHRs to uncover associations with COVID-19 infection^4,5^.

Longitudinal multi-center patient data in EHRs of over 20,000 COVID-19 patients (1803 hospitalized) from the Mayo Clinic (Rochester, Arizona, Florida) and associated health systems provide a unique opportunity to understand the relationship between comorbidities and COVID-19 complications^4^. While the structured EHR fields such as ICD codes are modestly informative, the true context of comorbidities and complications is buried in the millions of unstructured patient notes. In this study, we have leveraged ‘augmented curation’ of EHR notes in COVID-19 patients^4^ to map the relationships between complications, comorbidities, and outcomes in the hospitalized COVID-19 patients and non-COVID-19 hospitalized matched controls.

## Results

### Patient characteristics

**Fig. 1:**
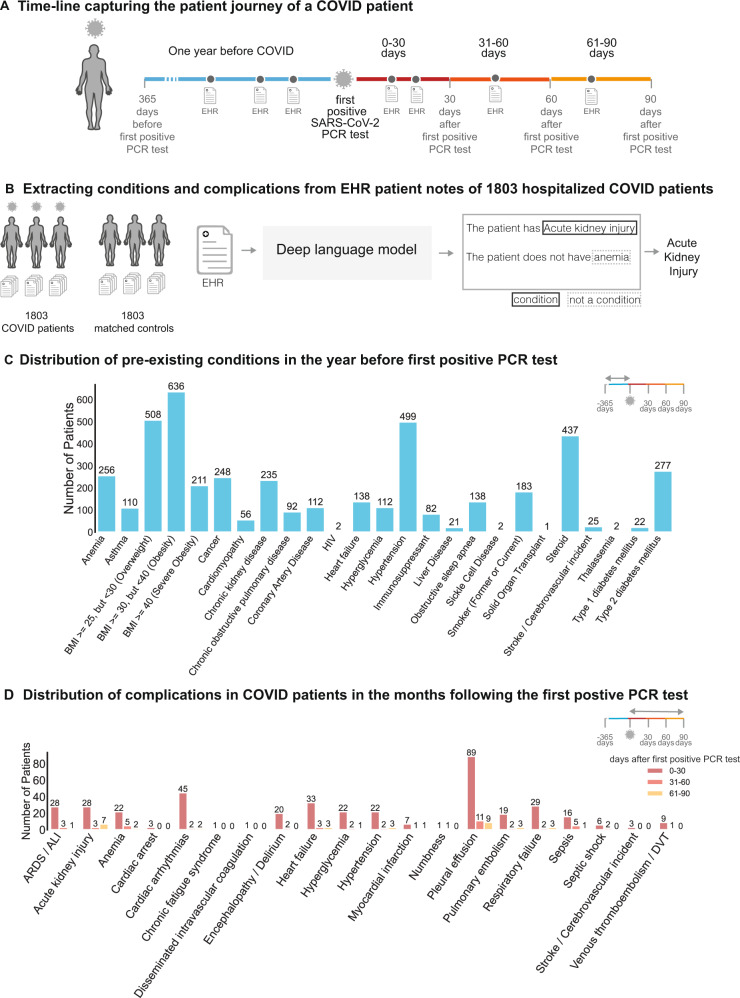
Study design and overview. **A** Relative timeline for each of the patients in the study, divided into the pre-COVID phase (1 year prior to the first positive PCR test), and the SARS-CoV-2 positive phase (90 days following the first positive PCR test). **B** Clinical notes from 1803 hospitalized COVID and non-COVID patients are analyzed with Bidirectional Encoder Representations from Transformers (BERT) model to extract the presence or absence of comorbidities and complications. **C** Distribution of pre-existing conditions in the first year before the first positive PCR test. **D** Distribution of complications at early (0–31 days) and late time points (31–60 days and 61–90 days) after the first positive PCR test.

**Fig. 2:**
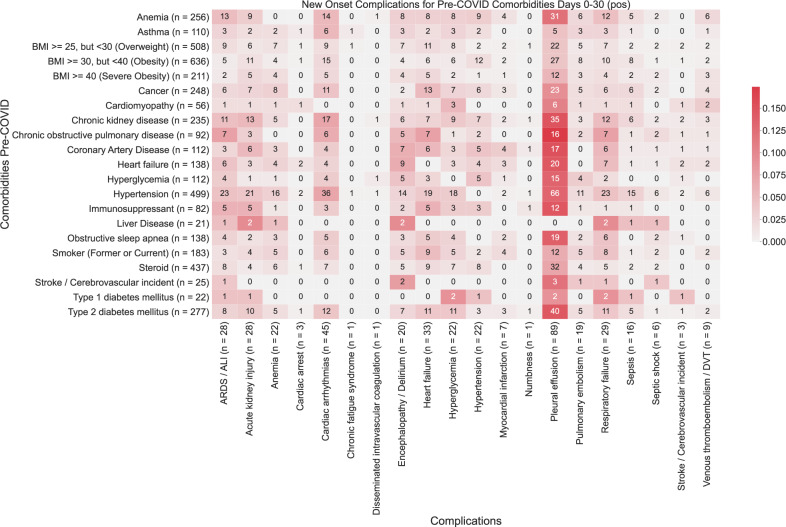
Heatmap showing associations between comorbidities and early-onset complications (0–30 days post-PCR) in COVID-19 patients. The color of each (comorbidity, complication) pair corresponds to (number of patients with the comorbidity that had the complication for the first time in the time window (0–30 days))/(total number of patients with the comorbidity). Darker shades of red correspond to higher rates of complications, and lighter shades of red correspond to lower rates of complications. A patient is determined to have comorbidity if it is recorded in their clinical notes with positive sentiment any time before their first positive PCR test. For each comorbidity row and for each complication column, the number of patients is shown in parentheses.

**Fig. 3:**
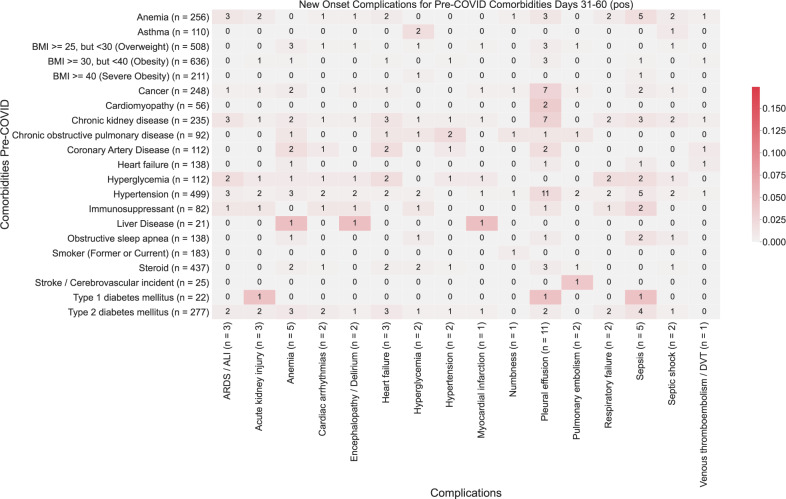
Heatmap showing associations between comorbidities and late-onset complications (31–60 days post-PCR) in COVID-19 patients. The color of each (comorbidity, complication) pair corresponds to (number of patients with the comorbidity that had the complication for the first time in the time window (31–60 days))/(total number of patients with the comorbidity). Darker shades of red correspond to higher rates of complications, and lighter shades of red correspond to lower rates of complications. A patient is determined to have comorbidity if it is recorded in their clinical notes with positive sentiment any time before their first positive PCR test. For each comorbidity row and for each complication column, the number of patients is shown in parentheses.

**Fig. 4:**
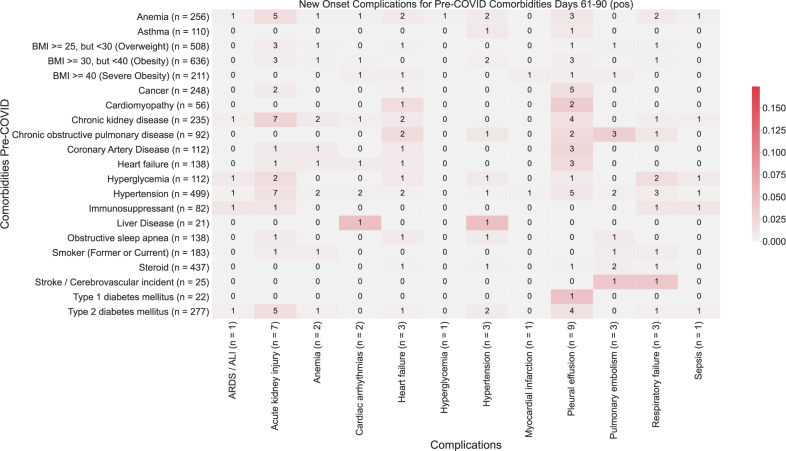
Heatmap showing associations between comorbidities and late-onset complications (61–90 days post-PCR) in COVID-19 patients. The number in each (comorbidity, complication) cell corresponds to the number of patients with the comorbidity that had the complication for the first time in the time window (61–90 days). The color of each (comorbidity, complication) pair corresponds to (number of patients with the comorbidity that had the complication for the first time in the time window (61–90 days))/(total number of patients with the comorbidity). Darker shades of red correspond to higher rates of complications, and lighter shades of red correspond to lower rates of complications. A patient is determined to have comorbidity if it is recorded in their clinical notes with positive sentiment any time before their first positive PCR test. For each comorbidity row and for each complication column, the number of patients is shown in parentheses.

1803 patients were hospitalized with a diagnosis of COVID-19 between March 12, 2020, and September 15, 2020. Using the date of the first positive SARS-CoV-2 PCR test, we analyzed the clinical notes of each patient in their pre-COVID-19 vs. the post-COVID-19 phase (Fig. 1A). Using deep language models (Fig. 1B), we extracted the 20 risk factors for COVID-19 severe illness reported by the CDC (Fig. 1C) and the 18 COVID-associated complications (Fig. 1D) in order to analyze their association in our cohort (Figs. 2–4).

In Table 1, we present the general characteristics of the study population. All age groups are included and, as expected from the severity of the disease in different age groups, more than 35.6% of the patients were over 65-year-old with only 3.7% under 19. Female, male and different ethnic origins of the US population are adequately represented. The most frequent comorbidities were hypertension (500 patients, 27.7%), type 2 diabetes mellitus (278 patients, 15.4%), obesity (227 patients, 12.6%), and cancer (254 patients, 14.1%), reflecting the most common causes of chronic diseases in the US. The most common COVID complications recorded were respiratory (ARDS, respiratory failure, pulmonary embolism), followed by cardiovascular (hypertension, myocardial infarction, arrhythmia, stroke), acute kidney injury, anemia, sepsis, and diabetic decompensation/hyperglycemia (Fig. 1D).

**Table 1:** General clinical characteristics of the study population.

Clinical covariate	COVID-positive cohort
Total number of patients	1803
*Age in years*	
0–19	66 (3.7%)
20–44	477 (26.5%)
45–64	616 (34.2%)
65–84	548 (30.4%)
85+	94 (5.2%)
*Sex*	
Female	874 (48.5%)
Male	929 (51.5%)
*Race*	
Asian	81 (4.5%)
Black/African American	211 (11.7%)
Native American	67 (3.7%)
White/Caucasian	1255 (69.6%)
Other	6 (0.3%)
Unknown	183 (10.1%)
*Ethnicity*	
Hispanic or Latino	291 (16.1%)
Not Hispanic or Latino	1452 (80.5%)
Unknown	60 (3.3%)
*Comorbidites*	
Anemia	270 (15.0%)
Asthma	113 (6.3%)
Cancer	254 (14.1%)
Cardiomyopathy	58 (3.2%)
Chronic Kidney Disease	233 (12.9%)
Chronic Obstructive Pulmonary Disease	93 (5.2%)
Coronary Artery Disease	111 (6.2%)
HIV	2 (0.1%)
Heart Failure	139 (7.7%)
Hyperglycemia	120 (6.7%)
Hypertension	500 (27.7%)
Liver Disease	24 (1.3%)
Neurologic Conditions	2 (0.1%)
Obesity	227 (12.6%)
Obstructive Sleep Apnea	133 (7.4%)
Pediatric Conditions	0 (0.0%)
Pregnancy	0 (0.0%)
Severe Obesity	16 (0.9%)
Sickle Cell Disease	2 (0.1%)
Solid Organ Transplant	1 (0.1%)
Stroke/Cerebrovascular Incident	25 (1.4%)
Thalassemia	2 (0.1%)
Type 1 Diabetes Mellitus	26 (1.4%)
Type 2 Diabetes Mellitus	278 (15.4%)

Clinical characteristics of all hospitalized COVID-19 positive patients in the Mayo Clinic EHR dataset. For each clinical covariate, the number of unique patients in the dataset is shown, with the percentage of the study of population in parentheses.

### Frequency of COVID-19 complications and association with underlying comorbidities

The main objective of our analysis was to identify associations between comorbidities and short-term (up to 30 days post-infection) and long-term (31–90 days post-infection) complications of COVID-19 infection. Here, we observe the majority of complications occur within the first month post-infection (Fig. 1D).

We identify multiple comorbidities that are associated with significantly higher rates of any complications in the early onset time period (days 0–30 post-PCR diagnosis). From this analysis, we validate that many of the CDC-reported risk factors for severe COVID-19 illness are associated with increased rates of early-onset COVID complications across multiple organ systems (Table 2). Among these, we identify hypertension (RR: 9.4, *p*-value: 2.9e−64) as the most significant risk factor followed by other cardiovascular chronic diseases (heart failure, coronary artery disease, cardiomyopathy), anemia (RR: 3.2, *p*-value: 9.8e−14), and chronic kidney disease (RR: 4.4, *p*-value: 1.5e−22), as the most significant predictors of clinical complication in early COVID-19 infection.

**Table 2:** Overall rates of early-onset complications stratified by comorbidity.

Comorbidity	Rate of new-onset complication in cohort with the comorbidity	Rate of new-onset complication in the cohort without the comorbidity	Relative risk [95% CI]	BH-adjusted *p*-value
Hypertension	113/500 (23%)	46/1303 (3.5%)	6.4 (4.6, 8.8)	1.6e−31
Chronic Kidney Disease	50/233 (21%)	109/1570 (6.9%)	3.1 (2.3, 4.2)	1.1e−09
Anemia	49/270 (18%)	110/1533 (7.2%)	2.5 (1.9, 3.5)	5.9e−07
Chronic Obstructive Pulmonary Disease	25/93 (27%)	134/1710 (7.8%)	3.4 (2.4, 5)	5.9e−07
Cancer	47/254 (19%)	112/1549 (7.2%)	2.6 (1.9, 3.5)	5.9e−07
Obesity	42/227 (19%)	117/1576 (7.4%)	2.5 (1.8, 3.5)	2.3e−06
Type 2 Diabetes Mellitus	48/278 (17%)	111/1525 (7.3%)	2.4 (1.7, 3.3)	2.6e−06
Heart Failure	27/139 (19%)	132/1664 (7.9%)	2.4 (1.7, 3.6)	1.8e−04
Coronary Artery Disease	23/111 (21%)	136/1692 (8%)	2.6 (1.8, 3.9)	2.0e−04
Obstructive Sleep Apnea	23/133 (17%)	136/1670 (8.1%)	2.1 (1.4, 3.2)	2.8e−03
Hyperglycemia	19/120 (16%)	140/1683 (8.3%)	1.9 (1.2, 3)	0.02
Type 1 Diabetes Mellitus	6/26 (23%)	153/1777 (8.6%)	2.7 (1.4, 5.5)	0.04
Asthma	17/113 (15%)	142/1690 (8.4%)	1.8 (1.2, 2.9)	0.04
Cardiomyopathy	9/58 (16%)	150/1745 (8.6%)	1.8 (1, 3.4)	0.16
Liver Disease	4/24 (17%)	155/1779 (8.7%)	1.9 (0.88, 4.8)	0.24
Severe Obesity	3/16 (19%)	156/1787 (8.7%)	2.1 (0.91, 6.1)	0.24
Neurologic Conditions	1/2 (50%)	158/1801 (8.8%)	5.7 (1.8, 18)	0.24
Stroke/Cerebrovascular Incident	1/25 (4%)	158/1778 (8.9%)	0.45 (0.14, 3.1)	0.96

In each row, we compare the rates of “early-onset” complications in cohorts of COVID-19 patients with and without comorbidities, during the time period (Days 0–30) relative to the PCR diagnosis date. To calculate the rates of new-onset complications, the numerator is the number of patients with any complication recorded in the clinical notes with positive sentiment during but not prior to the time period (see the “Methods” section for a full list of complications). The denominator is the number of patients without the complication recorded in the clinical notes with positive sentiment prior to Day 0. The columns are: (1) *Comorbidity*: Comorbidity that defines the cohorts, including chronic conditions which are risk factors for severe COVID-19 disease, (2) *Overall rate of new-onset complications in cohort with the comorbidity*: Overall rate of new-onset complications from Days 0–30 in the cohort of patients with the comorbidity. (3) *Overall rate of new-onset complications in the cohort without the comorbidity*: Overall rate of new-onset complications from Days 0–30 in the cohort of patients without the comorbidity, (4) *Relative risk [95% CI]*: (rate of complication in cohort with comorbidity)/(rate of complication in the cohort without comorbidity), along with the associated 95% confidence interval, (5) *BH-adjusted p-value:* Benjamini–Hochberg corrected *p*-value for the Fisher exact statistical significance test comparing the rates of overall complications in the cohorts of patients with and without the specified comorbidity.

### Respiratory complications

Pleural effusions are the most common early-onset complications: 4.9% of total patients (89 patients) within the first months (Fig. 1D). The primary risk factor for pleural effusion was hypertension (RR: 9.2, *p*-value: 2.4e−22) (Fig. 2; Supplementary Table 1). While the risk of new-onset of pleural effusion is reduced after a month, our data reveal persistent risk of pleural effusion beyond 30 days post-infection, particularly among patients with type 1 diabetes (5%, 1/22 patients) (Figs. 3 and 4).

Acute respiratory distress syndrome/acute lung injury is the second most frequent and is the most dreaded complication of severe COVID-19 infection (1.5% of total patients, 28 patients) (Fig. 1D). In the early stages of COVID infection (i.e. 0–30 days post-infection), ARDS/ALI was most significantly associated with hypertension (*p*-value: 4.2e−8). The other most significantly associated baseline comorbidities include anemia (*p*-value: 2.9e−4) and chronic kidney disease (*p*-value: 2.7e−3) (Fig. 2). In later stages of infection, we observe additional instances of ARDS/ALI, but at lower rates. Further, in later stages of infection, we fail to observe significant associations between baseline comorbidities and increased risk of new-onset of ARDS/ALI (Figs. 3, 4; Supplementary Table 1).

### Cardiovascular complications

Cardiac arrhythmia was the most common cardiovascular complication following COVID infection (2.5% of total patients, 45 patients) (Fig. 1D). Hypertension is by far the most important risk factor (RR: 21, *p*-value: 2.7e−19) (Supplementary Table 1). 7% (36 patients) of hypertensive patients present with this complication within the first 30 days (Fig. 2). But the risk among hypertensive declines to <1% (2 patients) for new-onset after one month post-infection (Figs. 3 and 4).

Early onset COVID heart failure is the second most common cardiovascular complication (1.8% of total patients, 33 patients) (Fig. 1D). It is primarily associated with coronary heart disease (RR: 7.3, *p*-value: 2.9e−3) and other cardiovascular risk factors (hypertension, anemia, type 2 diabetes, smoking) (Supplementary Table 1). But interestingly, cancer (RR: 5.1, *p*-value: 4.0e−4) and immunosuppression (RR: 4.5, *p*-value: 0.04) are also uncovered as significant risk factors. The cardiovascular complications examined in this study occur most frequently in days 0–30 post-infection (Fig. 2). Beyond 30 days, the risk of new-onset arrhythmia, hypertension, MI, PE/DVT dropped to less than 1% for all comorbidities (Figs. 3 and 4).

### Renal complications

Acute kidney injury is among the most common early-onset post-COVID complications (7%), (Fig. 1D) and is associated in our cohort mostly with hypertension (RR: 11, *p*-value: 1.2e−7), and chronic kidney disease (RR: 8.9, *p*-value: 4.1e−6) (Fig. 2; Supplementary Table 1). Specifically, we observe acute kidney injury in 1.6% (28 patients) of hospitalized COVID patients in aggregate in early infection. The risk of acute kidney injury is highest in the early stages of infection (i.e. 0–30 days post-infection), while there is a reduction in the new onset of acute kidney injury beyond 30 days (either 31–60 days or 61–90 days) (Figs. 3 and 4).

### Neurologic complications

Encephalopathy and delirium are commonly observed complications of COVID (c), which is most associated in our cohort with heart failure (RR: 11, *p*-value: 3.9e−5), hypertension (RR: 7.2, *p*-value: 3.3e−4), and coronary artery disease (RR: 9.2, *p*-value: 7.5e−4) (Supplementary Table 1). Further, the risk of encephalopathy and delirium was observed to be highest in early COVID infection (Figs. 3 and 4).

### Predictors of long-term complications of COVID-19 infection

We observe a substantial reduction in the frequency of new-onset of complications beyond 30 days post-infection (Fig. 1d). In the case of pleural effusion, which remains the most frequent complication, the prevalence decreases from 4.9% (89 patients) during the early onset time period (days 1–30) to <1% (20 patients) during the later onset time periods (days 31–90) (Figs. 2–4). In particular, patients with cardiomyopathy (2/56), chronic kidney disease (4/235), coronary artery disease (3/112), heart failure (3/138), and hypertension (5/499) appear to be more susceptible. Patients with liver disease, stroke, and type 1 diabetes also appear to be more susceptible to complications during days 31–90 post-infection (Figs. 3 and 4).

### Comparison to a propensity-matched population

In order to take into account the base rate of complications from in a hospitalized population, we generated a control population of COVID-negative patients that have been propensity matched on a number of clinical parameters including demographic covariates (age, sex, race, ethnicity) and comorbidities (the same set of 20 comorbidities). Using the same deep language models as described above, we extracted the 20 risk factors for COVID-19 severe illness reported by the CDC and the 18 COVID-associated complications in order to analyze their association among COVID-negative patients. As shown in Table 3, COVID-19 and COVID-19 negative cohorts were appropriately comparable. A subset of the comorbidities remained associated with early-onset complications higher than the baseline found in the COVID-19 negative cohort (Table 4). Hypertension remained the most frequent risk factor associated with early-onset respiratory failure (RR: 14, *p*-value: 1.0e−04) and heart failure (RR: 4.1, *p*-value: 0.02). Obesity appeared significantly associated with an increased risk of early-onset AKI (RR: 15, *p*-value: 0.02). Type 2 diabetes (RR: 21, *p*-value: 2.3e−03), anemia (RR: 12, *p*-value: 2.3e−03) and COPD (12% in COVID patients vs. none in non-COVID patients, *p*-value: 0.02) before infection were also associated with increased risk of respiratory complications. As discussed previously, late-onset complications were rare and not statistically associated with COVID-19 between the two cohorts.

**Table 3:** Characteristics of COVID-positive and COVID-negative patients.

Clinical covariate	COVID-positive cohort	1:1 propensity-matched COVID-negative cohort	Standardized mean difference (SMD)
Total number of patients	1803	1803	
*Age in years*			
0–19	66 (3.7%)	53 (2.9%)	0.04***
20–44	477 (26.5%)	455 (25.2%)	0.03***
45–64	616 (34.2%)	636 (35.3%)	0.02***
65–84	548 (30.4%)	579 (32.1%)	0.04***
85+	94 (5.2%)	78 (4.3%)	0.04***
*Sex*			
Female	874 (48.5%)	884 (49.0%)	0.01***
Male	929 (51.5%)	919 (51.0%)	0.01***
*Race*			
Asian	81 (4.5%)	83 (4.6%)	0.01***
Black/African American	211 (11.7%)	226 (12.5%)	0.03***
Native American	67 (3.7%)	48 (2.7%)	0.06***
White/Caucasian	1255 (69.6%)	1264 (70.1%)	0.01***
Other	6 (0.3%)	3 (0.2%)	0.03***
Unknown	183 (10.1%)	179 (9.9%)	0.01***
*Ethnicity*			
Hispanic or Latino	291 (16.1%)	315 (17.5%)	0.04***
Not Hispanic or Latino	1452 (80.5%)	1441 (79.9%)	0.02***
Unknown	60 (3.3%)	47 (2.6%)	0.04***
*Comorbidites*			
Anemia	270 (15.0%)	353 (19.6%)	0.12*
Asthma	113 (6.3%)	105 (5.8%)	0.02***
Cancer	254 (14.1%)	281 (15.6%)	0.04***
Cardiomyopathy	58 (3.2%)	72 (4.0%)	0.04***
Chronic Kidney Disease	233 (12.9%)	286 (15.9%)	0.08***
Chronic Obstructive Pulmonary Disease	93 (5.2%)	107 (5.9%)	0.03***
Coronary Artery Disease	111 (6.2%)	129 (7.2%)	0.04***
HIV	2 (0.1%)	4 (0.2%)	0.03***
Heart Failure	139 (7.7%)	169 (9.4%)	0.06***
Hyperglycemia	120 (6.7%)	135 (7.5%)	0.03***
Hypertension	500 (27.7%)	525 (29.1%)	0.03***
Liver Disease	24 (1.3%)	37 (2.1%)	0.06***
Neurologic Conditions	2 (0.1%)	3 (0.2%)	0.01***
Obesity	227 (12.6%)	252 (14.0%)	0.04***
Obstructive Sleep Apnea	133 (7.4%)	141 (7.8%)	0.02***
Pediatric Conditions	0 (0.0%)	1 (0.1%)	0.03***
Pregnancy	0 (0.0%)	1 (0.1%)	0.03***
Severe Obesity	16 (0.9%)	16 (0.9%)	0.00***
Sickle Cell Disease	2 (0.1%)	2 (0.1%)	0.00***
Solid Organ Transplant	1 (0.1%)	0 (0.0%)	0.03***
Stroke/Cerebrovascular Incident	25 (1.4%)	26 (1.4%)	0.00***
Thalassemia	2 (0.1%)	0 (0.0%)	0.05***
Type 1 Diabetes Mellitus	26 (1.4%)	14 (0.8%)	0.06***
Type 2 Diabetes Mellitus	278 (15.4%)	364 (20.2%)	0.12*

Covariates used for balancing were demographics (age, sex, race, ethnicity) and comorbidities. Columns are (1) Clinical covariate, (2) *COVID-positive cohort*: the proportion of patients with the covariate, (3) *1:1 propensity-matched COVID-negative cohort*: the proportion of patients with the covariate, (4) *Standardized mean difference (SMD)*: the standardized mean difference (or Cohen’s *d*) between the COVID-positive and 1:1 propensity-matched COVID-negative cohort. The SMD is a measure of difference between the matched cohorts; we denote SMD < 0.1 to be “highly balanced” and indicate these values with ***; we denote 0.1 ≤ SMD < 0.25 to be moderately balanced and indicate these with *.

**Table 4:** Rates of early onset complications stratified by comorbidity.

Complication	Comorbidity	Rate of new onset complication in COVID-positive cohort with the Comorbidity	Rate of new onset complication in COVID-negative cohort with the Comorbidity	Relative Risk [95% CI]	BH-adjusted *p*-value
Acute respiratory distress syndrome/Acute lung injury	Hypertension	27/489 (5.5%)	2/521 (0.38%)	14 (3.2, 43)	1.0E-04
Respiratory failure	Hypertension	33/472 (7%)	5/518 (0.97%)	7.2 (2.7, 16)	1.0E-04
Respiratory failure	Type 2 diabetes mellitus	15/263 (5.7%)	1/360 (0.28%)	21 (2.7, 75)	2.3E-03
Respiratory failure	Anemia	17/250 (6.8%)	2/343 (0.58%)	12 (2.6, 36)	2.3E-03
Respiratory failure	Chronic obstructive pulmonary disease	10/81 (12%)	0/105 (0%)	∞ (1.6, ∞)	0.02
Heart failure	Hypertension	26/384 (6.8%)	7/420 (1.7%)	4.1 (1.7, 8.6)	0.02
Acute kidney injury	Obesity	12/190 (6.3%)	1/241 (0.41%)	15 (2, 57)	0.02

In each row, we compare the rates of “early onset” (i.e. days 0-30 relative to PCR test date) complications in cohorts of COVID-positive and matched COVID-negative patients with particular comorbidities. For the positive patients, PCR test date is the first positive PCR test; for negative patients it is the first negative PCR test. Rows are sorted first by complication, and second by statistical significance. Only those comorbidity-complication pairs which showed a statistically significant difference (BH-adjusted p-value <= 0.05) in rates of new onset complications are shown. The columns are: (1) *Complication*: Complication phenotype that is used to define the rates, including phenotypes associated with severe COVID-19 disease, (2) *Comorbidity*: Comorbidity that defines the cohorts, including chronic conditions which are risk factors for severe COVID-19 disease, (3) *Rate of new onset complication in COVID-positive cohort with comorbidity*: Rate of complication from Days 0-30 in the cohort of patients with the comorbidity. (4) *Rate of new onset complication in matched COVID-negative cohort with comorbidity*: Rate of complication from Days 0-30 in the cohort of COVID-negative patients with the comorbidity, (5) *Relative risk [95% CI*.*]*: (Rate of complication in COVID-positive cohort with comorbidity) / (Rate of complication in COVID-negative cohort with comorbidity), along with the associated 95% confidence interval, (6) *BH-adjusted p-value*: Benjamin-Hochberg corrected p-value for the Fisher exact statistical significance test comparing the rates of the specified complications in the cohorts of COVID-positive vs COVID-negative patients with the comorbidity.

## Discussion

In the present study we have set out to understand the relationship between baseline comorbidities and clinical complications over the course of COVID-19 infection. Here, we leverage natural language processing of unstructured patient notes from 1803 patients hospitalized with COVID-19 in the Mayo Clinic health system.

While it stands to reason that individuals with poorer health status and multiple underlying comorbidities will experience worse outcomes during COVID-19 infection, our study reveals that not all risk factors are created equal and are associated with different complications. Previous studies have begun to uncover numerous factors associated with increased risk of more severe COVID-19 infection^6–12^ including hypertension, chronic kidney disease, type 2 diabetes, cardiovascular disease, and malignancy. In general, these studies have examined risk of severe COVID-19 infection but have not examined the relationship between baseline comorbidities and risk of specific complications. Furthermore, our study has leveraged augmented curation to accelerate the mapping of the comorbidities and COVID-associated complications.

In our analysis, we observe that hypertension is the single most significant risk factor among all examined complications with exception of deep vein thrombosis. This is consistent with previous studies, where patients with baseline hypertension have been reported to have higher risk of more severe COVID-19 disease^7,8^. Specifically, our data suggest that a recent history of hypertension is the strongest predictor of ARDS, the most significant and life-threatening complication of COVID-19, among hospitalized COVID-19 patients, similar to previous observations^10^. We further observed anemia, chronic kidney disease, immunosuppression, coronary artery disease and hyperglycemia to be associated with increased rates of ARDS. Our analysis also uncovered unexpected associations, including associations between history of cancer and immunosuppression with heart failure following COVID-19 infection.

Our data also highlight the temporal relationship between baseline health status and complications throughout COVID-19 infection. For example, cancer, obesity, and obstructive sleep apnea are associated with higher rates of short-term complications (days 0–30 post-PCR test), but not with late-onset complications. While many comorbidities are chronic (e.g. cancer, obesity, coronary artery disease, and chronic kidney disease), others are amenable to short-term intervention, suggesting that tight control of modifiable risk factors might limit the risk of complication due to COVID-19 infection. For example, controlling hypertension, smoking cessation, treating anemia, and having tight glycemic control might reduce the rate of cardiovascular complications in the early stages of COVID-19.

Many of the comorbidities examined likely influence the development of complications, even in the absence of COVID-19 infection. For example, we do not observe new-onset pleural effusion among patients with pre-existing liver disease. It is possible that this is related to previous incidence of pleural effusion among patients with liver disease^13^. While our analyses are limited to hospitalized COVID-19 patients, which biases our data towards patients with more severe disease, this enables improved risk stratification for patients most likely to develop serious complications. We have further explored the rate of clinical complications in a control population of hospitalized COVID-negative patients to establish baseline complication rates within a hospitalized population.

At present, our analysis does not account for the co-dependent relationships between comorbidities or between complications. In many cases, individual patients likely have multiple complications, which can obscure the interpretation of data, particularly at later time points where we observe fewer events. Another limitation of this study is that we employ a relatively broad inclusion criteria (all COVID-19 hospitalized patients in the Mayo Clinic EHR system), so more focused observational studies with stricter eligibility criteria would be required to obtain robust conclusions about a particular patient subpopulation. One potential data limitation may be that the comorbidities of the patient population are not fully captured by the EHR database, because the patients may have received care at other institutions prior to receiving treatment at the Mayo Clinic. However, we expect that most of the major comorbidities for these patients will be captured because all of these patients received hospital care for COVID-19 through Mayo Clinic Health Systems. Additionally, it is possible that many of the late-stage complications arise directly from baseline comorbidities rather than a direct result of COVID-19 infection. This study can be leveraged for the development of controlled trials to identify appropriate prophylactic or therapeutic interventions for high-risk COVID-19 patients, particularly among hospitalized patients. Future analyses will focus on creation of a multivariate model to enable risk prediction of post-COVID complications^14^.

Given the richness and complexity of information present in the clinical notes, there are multiple promising avenues for future research to develop the natural language processing (NLP) models further. For example, Bayesian modeling can be used to aggregate the sentence-level BERT sentiment predictions into robust patient-level sentiment predictions. Although the sentence-level accuracy of the BERT model is currently 98.0% (see the “Methods” section), there is potential for improvement if we aggregate all of the sentences from each patient to determine the predictions. Interpreting potentially conflicting sentences in a single clinical note or in a set of clinical notes for a patient is a challenge when applying NLP methods to the unstructured text in the EHR, and is an important area for future study. For example, sentence-level models may be validated by expert determination at the document-level and patient-level and compared against models which incorporate additional contextual information. Furthermore, NLP methods may be developed to determine other health indicators from the clinical notes such as disease severity and quality of life.

There are also numerous promising clinical directions for future research. For example, we may discover novel predictors of COVID-19 complications by exploring comorbidities which are not identified as risk factors of severe COVID-19 disease by the CDC. There have been initial studies of patients with long-term complications from COVID-19 (aka “long-COVID”)^15^, and mapping risk factors to complications in long-COVID patients is a particularly interesting area for follow-up research^16^. Since this study was restricted to patients in a single academic medical center, studies in other medical centers would be valuable to validate the clinical findings and see how the results generalize to different patient populations (e.g. non-hospitalized patients, non-white patients). One of the major contributions of this work is the development of the NLP methodology to make exploration of comorbidities and complications in the unstructured clinical record more efficient. We hope that this research can pave the way for future observational studies leveraging the rich diversity of clinical phenotypes in unstructured notes to explore a broad range of scientific questions.

## Methods

### Institutional Review Board (IRB)

This retrospective research was conducted under IRB 20-003278: “Study of COVID-19 patient characteristics with augmented curation of EHRs to inform strategic and operational decisions”. The study was deemed exempt by the Mayo Clinic Institutional Review Board and waived from consent. For further information regarding the Mayo Clinic Institutional Review Board (IRB) policy, and its institutional commitment, membership requirements, review of research, informed consent, recruitment, vulnerable population protection, biologics, and confidentiality policy, please refer to www.mayo.edu/research/institutional-review-board/overview.

### Study design

This was an observational study of 1803 hospitalized COVID-19 positive patients (positive PCR for SARS-CoV-2) in the Mayo Clinic electronic health record (EHR) database from March 12, 2020 to September 15, 2020. The sample size was determined based upon the number of patients in the Mayo Clinic EHR database at the time of the study in order to maximize the power of the downstream statistical tests. Patients who declined to give research authorization were excluded from the study. No patients were excluded on the basis of age, sex, ethnicity, or other clinical parameters. In order to compare rates of comorbidities and complications against a baseline, a control cohort of 94,953 COVID-19 negative hospitalized patients in the Mayo Clinic EHR database during the same time period was considered. Propensity score matching^17^ was performed in order to control for potential confounding factors when comparing the COVID-positive and COVID-negative cohorts. First, propensity scores for each of the patients in the two cohorts were computed by fitting a logistic regression model (using scikit-learn v0.20.3 in python 3.6.8) as a function of the covariates, including demographic variables (age, sex, race, ethnicity) and comorbidities. Next, patients from the COVID-positive and COVID-negative cohorts were matched using a 1:1 matching ratio and a heuristic caliper of 0.1 × pooled standard deviation^18^. Prior to matching, there were 1803 patients in the COVID-positive cohort, and there were 94,953 patients in the COVID-negative cohort. Matched controls were found for all COVID-positive patients, giving us a matched COVID-negative cohort of 1803 patients. An overview of the clinical characteristics of the study population is provided in Tables 1 and 4. No follow-up clinical experiments were performed to verify the findings in this study. Blinding was not relevant due to the observational nature of this study.

### Comorbidities and complications

For comorbidities, we considered 21 risk factors for COVID-19 severe illness reported by the CDC^19^, including: anemia, asthma, BMI between 25–30 (overweight), BMI between 30–40 (obese), BMI ≥ 40 (severe obesity), cancer, cardiomyopathy, chronic kidney disease (CKD), chronic obstructive pulmonary disease (COPD), coronary artery disease (CAD), heart failure (HF), hyperglycemia, hypertension, immunosuppressant medication usage, liver disease, neurologic conditions, obstructive sleep apnea (OSA), smoker (former or current), steroid medication usage, type 1 diabetes mellitus (T1D), type 2 diabetes mellitus (T2D). We also note that bone marrow transplant, HIV/AIDS, pediatric conditions, pregnancy, sickle cell disease, solid organ transplant, and thalassemia were also considered, but were not included in the analysis because fewer than 20 patients had each of these comorbidities.

For complications, we considered 20 COVID-associated complications collected by the Society for Critical Care Medicine (SCCM) Viral Infection and Respiratory Illness Universal Study (VIRUS) data registry^20^ and analyzed in follow-up observational studies^21^, including: acute respiratory distress syndrome/acute lung injury (ARDS/ALI), acute kidney injury (AKI), anemia, cardiac arrest, cardiac arrhythmias, chronic fatigue syndrome, disseminated intravascular coagulation (DIC), heart failure, hyperglycemia, hypertension, myocardial infarction (MI), pleural effusion, pulmonary embolism (PE), respiratory failure, sepsis, septic shock, stroke/cerebrovascular incident, venous thromboembolism/deep vein thrombosis (VTE/DVT), delirium/encephalopathy, and numbness.

### Augmented curation to identify comorbidities and complications in clinical notes

An augmented curation approach was used to classify the sentiment of phenotypes mentioned in the clinical notes across the Mayo Clinic healthcare system. The Mayo Clinic EHR system relies upon Epic for electronic management of health records which is used by providers across its campuses. For this analysis, we considered a database of 2.0 million clinical notes covering all of the unstructured text written, typed, or dictated into the Epic system during the course of clinical care for the study population and propensity-matched controls, including but not limited to: progress notes, discharge summaries, inpatient notes, outpatient notes, and telephone call transcripts. This corresponds to an average of 567 notes per patient for the study population and the propensity-matched cohort which each includes 1803 patients. We note that radiology reports and pathology reports were not included in this dataset. In Supplementary Fig. 1, we provide the frequency distribution showing the number of notes per patient. For each day that a patient has noted in their electronic health record, the average number of notes per day is 3.6 (standard deviation: 5.9). In order to identify patients with complications and comorbidities from the clinical notes, we used a neural network-based sentiment model, which we describe next.

A BERT-based neural network was applied to identify phenotypes of interest in the clinical notes of the study population^4^. The model was previously developed to classify the sentiment of general phenotypes^4^ and thrombotic event phenotypes^5^ in the encounter notes of COVID-19 patients. The categories of this classification model for each comorbidity and complication include: Yes (confirmed diagnosis), No (ruled out a diagnosis), Maybe (possibility of disease), and Other (alternate context, e.g. family history of disease). This model was trained using nearly 250 different phenotypes and 18,490 sentences and achieves 93.6% overall accuracy and over 95% precision and recall for Yes/No sentiment classification^4^. For this study, the phenotypes of interest included a specific list of comorbidities and complications along with their synonyms. The comorbidities list consists of asthma, cancer, chronic kidney disease, chronic obstructive pulmonary disease, obesity, obstructive sleep apnea, type 1 diabetes mellitus, and type 2 diabetes mellitus. The complications list includes acute respiratory distress syndrome/acute lung injury, acute kidney injury, anemia, cardiac arrest, cardiac arrhythmias, disseminated intravascular coagulation, heart failure, hyperglycemia, hypertension, myocardial infarction, pleural effusion, pulmonary embolism, respiratory failure, sepsis, septic shock, stroke/cerebrovascular incident, venous thromboembolism/deep vein thrombosis.

We used this model to classify the sentiment for all of the above phenotypes in all of the clinical notes for each patient in the time periods considered in this study. Only sentences containing a phenotype with a positive sentiment (labeled “Yes” by the model) with a confidence of 0.95 or above were deemed positive sentiment associations. In this analysis, repeated sentences for the same patient were ignored. For each patient, in each time period, we consider the phenotype to be present if there are at least three positive mentions in the clinical notes, regardless of the number of negative mentions. As a result, patients who were recorded as negative for a particular phenotype at some time may be classified as positive for the phenotype-based upon the other clinical notes during the time period.

The model used to curate complications/comorbidities was initially validated, as previously described^4^. In order to validate the augmented curation model for the present set of phenotypes of complications/comorbidities, we manually labeled a set of 2404 randomly selected sentences from the clinical notes containing the phenotypes. This validation set of 2404 sentences was derived from ~2000 (0.1%) unique notes in the dataset of 2.0 million notes for the study population and propensity-matched cohort. For each phenotype, the sentences in this validation set were selected by randomly sampling the clinical notes from the 1803 COVID-19-positive patients in the study population. The true positive, true negative, false positive, and false-negative rates are reported in Supplementary Table 2. We note that for most of the phenotypes, the majority class label is “Yes”, indicating a confirmed diagnosis of the phenotype. Overall, the out-of-sample precision, recall, and accuracy values were 98.0%, 98.2%, and 96.6%, respectively. These validation results are consistent with the validation results from a previous study using a phenotype sentiment model to identify cases of thrombotic events from unstructured clinical notes in the Mayo Clinic EHR^5^.

### Comorbidities—complications association calculations

A patient was determined to have a clinical phenotype (the comorbidity or complication, in question) if the clinical phenotype or synonyms were mentioned (with positive sentiment) within that patients’ EHRs, as described above. For comorbidities, the mention must have occurred within a note at any point in the patient history prior to the patient’s first positive COVID-19 PCR test. Patients were only considered if they had at least one note within the Mayo Clinic EHR system dated before days −31 relative to their first positive PCR test.

For the patients included in this study, we stratified the rates of new-onset complications by comorbidities. For each comorbidity, e.g. chronic kidney disease, we compare the rates of “new-onset” complications in cohorts of COVID-19 patients with and without chronic kidney disease. To calculate the rate of a new-onset complication, e.g. acute kidney injury (AKI), the numerator is the number of patients with AKI recorded in the clinical notes (with positive sentiment) during but not prior to the time period. The denominator is the number of patients without AKI recorded in the clinical notes with positive sentiment prior to the time period.

### Statistical analysis

To determine the relationship between individual comorbidities/pre-existing conditions and complications of COVID-19 infection, we performed a two-sided Chi-square test to compare the frequency of complications in patients with and without a given co-morbidity. Statistical significance tests were run using the software package scipy v1.5.4 in Python. The software package scikit-learn v0.20.3 in Python was used to train logistic regression models for the propensity score matching analysis.

### Reporting summary

Further information on research design is available in the Nature Research Reporting Summary linked to this article.

## Supplementary information

Supplementary Information

Reporting Summary

## Data Availability

Reasonable requests for de-identified data made to the corresponding author will be reviewed and processed by the Mayo Clinic institutional review board upon publication of this manuscript.

## Code availability

The code for the statistical analyses will be made available upon request to the corresponding author. All code was written in Python 3.6.8 using the following open source software packages: scikit-learn v0.20.3, scipy v1.5.4.
