# Supplementary material for: Mapping each pre-existing condition’s association to short-term and long-term COVID-19 complications
Source: NPJ Digit Med. 2021 Jul 27;4:117. doi: 10.1038/s41746-021-00484-7 (PMC8316410; doi:10.1038/s41746-021-00484-7)
Supplement: Supplementary file 1 — Supplementary Information [file 41746_2021_484_MOESM1_ESM.pdf]

Supplementary Material

Supplementary Figure 1: Distribution of the number of clinical notes per patient in the Mayo Clinic EHR dataset. In this histogram, we show the number of notes per patient for the 1,803 patients in the study population along with the propensity matched controls. For each patient, we add up the number of notes that are present in the Mayo Clinic Health System EHR dataset from the beginning of their electronic health record to September 15, 2020. The summary statistics for the distribution of clinical notes are: (mean: 567, standard deviation: 861, 25th percentile: 44, 50th percentile: 260, 75th percentile: 737, min: 0, max: 10,472).

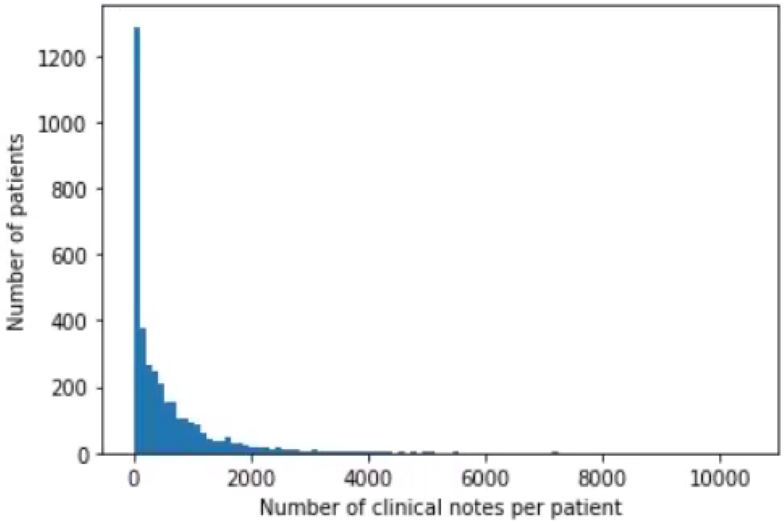

Supplementary Table 1: Rates of early onset complications stratified by comorbidity In each row, we compare the rates of “early onset” complications in cohorts of COVID-19 patients with and without comorbidities, during the time period (Days 0-30) relative to the PCR diagnosis date`. To calculate the rates of new onset complications, the numerator is the number of patients with the complication recorded in the clinical notes with positive sentiment during but not prior to the time period. The denominator is the number of patients without the complication recorded in the clinical notes with positive sentiment prior to the time period. Rows with cardiovascular complications are highlighted in **light red**, and rows with respiratory complications are highlighted in **light blue**. Rows are sorted first by complication, and second by statistical significance. The columns are: **(1) Complication:** Complication phenotype that is used to define the rates, including phenotypes associated with severe COVID-19 disease, **(2) Comorbidity:** Comorbidity that defines the cohorts, including chronic conditions which are risk factors for severe COVID-19 disease, **(3) Rate of new onset complication in cohort with the comorbidity:** Rate of complication from Days 0-30 in the cohort of patients with the comorbidity. **(4) Rate of new onset complication in cohort without the comorbidity:** Rate of complication from Days 0-30 in the cohort of patients without the comorbidity, **(5) Relative risk [95% C.I.]:** (Rate of complication in cohort with comorbidity) / (Rate of complication in cohort without comorbidity), along with the associated 95% confidence interval, **(6) BH-adjusted p-value:** Benjamin-Hochberg corrected p-value for the Fisher exact statistical significance test comparing the rates of the specified complications in the cohorts of patients with and without the specified comorbidity.

Complication	Comorbidity	Rate of new onset complication in cohort with the Comorbidity	Rate of new onset complication in cohort without the Comorbidity	Relative Risk [95% CI]	BH-adjusted p-value
Acute respiratory distress syndrome / Acute lung injury	Hypertension	27/500 (5.4%)	8/1303 (0.61%)	8.8 (3.9, 18)	1.8E-07
Acute respiratory distress syndrome / Acute lung injury	Chronic obstructive pulmonary disease	9/93 (9.7%)	26/1710 (1.5%)	6.4 (3.2, 13)	1.4E-03
Acute respiratory distress syndrome / Acute lung injury	Heart failure	10/139 (7.2%)	25/1664 (1.5%)	4.8 (2.4, 9.8)	4.4E-03
Acute respiratory distress syndrome / Acute lung injury	Chronic kidney disease	11/233 (4.7%)	24/1570 (1.5%)	3.1 (1.6, 6.3)	0.04
Acute kidney injury	Hypertension	28/500 (5.6%)	12/1303 (0.92%)	6.1 (3.1, 11)	2.4E-06
Acute kidney injury	Chronic kidney disease	16/233 (6.9%)	24/1570 (1.5%)	4.5 (2.5, 8.3)	5.6E-04
Acute kidney injury	Obesity	12/227 (5.3%)	28/1576 (1.8%)	3 (1.6, 5.8)	0.03
Anemia	Hypertension	19/500 (3.8%)	7/1303 (0.54%)	7.1 (2.9, 16)	9.6E-05

Cardiac arrhythmias	Hypertension	44/500 (8.8%)	13/1303 (1%)	8.8 (4.7, 16)	1.8E-12
Cardiac arrhythmias	Chronic kidney disease	19/233 (8.2%)	38/1570 (2.4%)	3.4 (2, 5.8)	1.4E-03
Cardiac arrhythmias	Anemia	18/270 (6.7%)	39/1533 (2.5%)	2.6 (1.5, 4.5)	0.02
Cardiac arrhythmias	Type 2 diabetes mellitus	17/278 (6.1%)	40/1525 (2.6%)	2.3 (1.4, 4.1)	0.05
Heart failure	Hypertension	26/500 (5.2%)	12/1303 (0.92%)	5.6 (2.8, 11)	1.0E-05
Heart failure	Cancer	14/254 (5.5%)	24/1549 (1.5%)	3.6 (1.9, 6.8)	7.2E-03
Hyperglycemia	Hypertension	26/500 (5.2%)	10/1303 (0.77%)	6.8 (3.2, 13)	2.4E-06
Hyperglycemia	Type 2 diabetes mellitus	14/278 (5%)	22/1525 (1.4%)	3.5 (1.8, 6.7)	9.5E-03
Hyperglycemia	Cancer	12/254 (4.7%)	24/1549 (1.5%)	3 (1.6, 6.1)	0.03
Hyperglycemia	Chronic kidney disease	11/233 (4.7%)	25/1570 (1.6%)	3 (1.5, 6)	0.05
Hypertension	Hypertension	0/500 (0%)	28/1303 (2.1%)	0 (0.0028, 0.75)	3.9E-03
Hypertension	Hyperglycemia	7/120 (5.8%)	21/1683 (1.2%)	4.7 (2.2, 11)	0.03
Hypertension	Heart failure	7/139 (5%)	21/1664 (1.3%)	4 (1.8, 9.4)	0.05
Pleural effusion	Type 2 diabetes mellitus	15/278 (5.4%)	16/1525 (1%)	5.1 (2.6, 10)	5.6E-04
Pleural effusion	Obesity	13/227 (5.7%)	18/1576 (1.1%)	5 (2.5, 10)	1.4E-03
Pleural effusion	Hypertension	19/500 (3.8%)	12/1303 (0.92%)	4.1 (2, 8.2)	3.3E-03
Pleural effusion	Heart failure	9/139 (6.5%)	22/1664 (1.3%)	4.9 (2.4, 11)	7.2E-03
Pleural effusion	Chronic obstructive pulmonary disease	7/93 (7.5%)	24/1710 (1.4%)	5.4 (2.5, 12)	0.01
Pleural effusion	Anemia	12/270 (4.4%)	19/1533 (1.2%)	3.6 (1.8, 7.3)	0.02
Pleural effusion	Coronary Artery Disease	7/111 (6.3%)	24/1692 (1.4%)	4.4 (2.1, 10)	0.03
Pulmonary embolism	Anemia	8/270 (3%)	6/1533 (0.39%)	7.6 (2.7, 20)	6.9E-03
Pulmonary embolism	Cancer	7/254 (2.8%)	7/1549 (0.45%)	6.1 (2.2, 17)	0.02
Respiratory failure	Hypertension	33/500 (6.6%)	11/1303 (0.84%)	7.8 (3.9, 15)	1.3E-08
Respiratory failure	Heart failure	13/139 (9.4%)	31/1664 (1.9%)	5 (2.8, 9.4)	5.6E-04

Respiratory failure	Chronic obstructive pulmonary disease	10/93 (11%)	34/1710 (2%)	5.4 (2.9, 11)	1.4E-03
Respiratory failure	Anemia	17/270 (6.3%)	27/1533 (1.8%)	3.6 (2, 6.5)	2.5E-03
Respiratory failure	Obesity	14/227 (6.2%)	30/1576 (1.9%)	3.2 (1.8, 6)	0.01
Respiratory failure	Chronic kidney disease	14/233 (6%)	30/1570 (1.9%)	3.1 (1.7, 5.9)	0.01
Respiratory failure	Type 2 diabetes mellitus	15/278 (5.4%)	29/1525 (1.9%)	2.8 (1.6, 5.2)	0.03
Respiratory failure	Obstructive sleep apnea	9/133 (6.8%)	35/1670 (2.1%)	3.2 (1.7, 6.7)	0.04
Respiratory failure	Coronary Artery Disease	8/111 (7.2%)	36/1692 (2.1%)	3.4 (1.7, 7.2)	0.05
Sepsis	Hypertension	18/500 (3.6%)	3/1303 (0.23%)	16 (4.4, 43)	3.1E-06
Sepsis	Cancer	9/254 (3.5%)	12/1549 (0.77%)	4.6 (2, 11)	0.02
Sepsis	Anemia	9/270 (3.3%)	12/1533 (0.78%)	4.3 (1.9, 9.9)	0.03
Septic shock	Hypertension	7/500 (1.4%)	2/1303 (0.15%)	9.1 (1.9, 33)	0.03
Venous thromboembolism / DVT	Anemia	7/270 (2.6%)	7/1533 (0.46%)	5.7 (2.1, 15)	0.03
Venous thromboembolism / DVT	Hypertension	9/500 (1.8%)	5/1303 (0.38%)	4.7 (1.6, 13)	0.05
Venous thromboembolism / DVT	Obesity	6/227 (2.6%)	8/1576 (0.51%)	5.2 (1.9, 15)	0.05

Supplementary Table 2: Sentence-level validation of BERT model to classify phenotype sentiment. The columns are **(1) Phenotype:** disease token identified in the sentence, **(2) TP (true positives):** count of sentences in which the BERT model correctly identified the sentiment as 'Yes', **(3) TN (true negatives):** count of sentences in which the BERT model correctly identified the sentiment as not 'Yes', **(4) FP (false positives):** count of sentences in which the BERT model incorrectly identified the sentiment as 'Yes', **(5) FN: (false negatives):** count of sentences in which the BERT model incorrectly identified the sentiment as not 'Yes', **(6) Precision:** precision of the BERT model, equal to TP/(TP+FP), **(7) Recall:** recall of the BERT model, equal to TP/(TP+FN), **(8) Accuracy:** accuracy of the BERT model, equal to (TP+TN)/(TP+TN+FP+FN).

Phenotype	TP	FP	FN	TN	Precision	Recall	Accuracy
Acute kidney injury	93	3	2	0	0.969	0.979	0.949
Anemia	97	5	1	5	0.951	0.99	0.945
Acute respiratory distress syndrome / acute lung injury	98	0	1	4	1	0.99	0.991
Asthma	89	3	1	11	0.968	0.989	0.962
Cancer	80	1	5	19	0.988	0.942	0.943
Cardiac arrest	63	1	10	4	0.985	0.864	0.859
Cardiac arrhythmias	96	0	1	6	1	0.99	0.991
Chronic kidney disease	104	5	0	0	0.955	1	0.955
Chronic obstructive pulmonary disease	1	0	0	0	1	1	1
Disseminated intravascular coagulation	18	2	6	50	0.9	0.75	0.895
Heart failure	90	4	1	9	0.958	0.99	0.952
Hyperglycemia	96	2	1	12	0.98	0.99	0.973
Hypertension	99	1	1	3	0.99	0.99	0.981
Myocardial infarction	85	0	1	14	1	0.989	0.99
Obesity	98	0	2	0	1	0.98	0.98
Obstructive sleep apnea	92	1	0	7	0.99	1	0.99

Pleural effusion	56	0	1	28	1	0.983	0.989
Pulmonary embolism	50	1	2	46	0.981	0.962	0.97
Respiratory failure	111	0	0	0	1	1	1
Sepsis	95	7	1	18	0.932	0.99	0.934
Septic shock	80	1	0	8	0.988	1	0.989
Stroke / Cerebrovascular incident	81	1	0	12	0.988	1	0.99
Type 1 diabetes mellitus	93	3	0	1	0.969	1	0.97
Type 2 diabetes mellitus	106	0	0	1	1	1	1
Venous thromboembolism / deep vein thrombosis	79	2	2	14	0.976	0.976	0.959
**Overall**	**2050**	**43**	**39**	**272**	**0.980**	**0.982**	**0.966**
