# Supplementary material for: Mapping each pre-existing condition’s association to short-term and long-term COVID-19 complications
Source: NPJ Digit Med. 2021 Jul 27;4:117. doi: 10.1038/s41746-021-00484-7 (PMC8316410; doi:10.1038/s41746-021-00484-7)
Supplement: Supplementary file 2 — Reporting Summary [file 41746_2021_484_MOESM2_ESM.pdf]

nature research

Corresponding author(s): Venky Soundararajan

Last updated by author(s): Apr 14, 2021

Reporting Summary

Nature Research wishes to improve the reproducibility of the work that we publish. This form provides structure for consistency and transparency in reporting. For further information on Nature Research policies, see our Editorial Policies and the Editorial Policy Checklist.

Statistics

For all statistical analyses, confirm that the following items are present in the figure legend, table legend, main text, or Methods section.

n/a	Confirmed	
☐	☒	The exact sample size (*n*) for each experimental group/condition, given as a discrete number and unit of measurement
☒	☐	A statement on whether measurements were taken from distinct samples or whether the same sample was measured repeatedly
☐	☒	The statistical test(s) used AND whether they are one- or two-sided<br>*Only common tests should be described solely by name; describe more complex techniques in the Methods section.*
☐	☒	A description of all covariates tested
☐	☒	A description of any assumptions or corrections, such as tests of normality and adjustment for multiple comparisons
☒	☐	A full description of the statistical parameters including central tendency (e.g. means) or other basic estimates (e.g. regression coefficient) AND variation (e.g. standard deviation) or associated estimates of uncertainty (e.g. confidence intervals)
☐	☒	For null hypothesis testing, the test statistic (e.g. *F*, *t*, *r*) with confidence intervals, effect sizes, degrees of freedom and *P* value noted<br>*Give P values as exact values whenever suitable.*
☒	☐	For Bayesian analysis, information on the choice of priors and Markov chain Monte Carlo settings
☒	☐	For hierarchical and complex designs, identification of the appropriate level for tests and full reporting of outcomes
☐	☒	Estimates of effect sizes (e.g. Cohen's *d*, Pearson's *r*), indicating how they were calculated

Our web collection on statistics for biologists contains articles on many of the points above.

Software and code

Policy information about availability of computer code

Data collection	The Mayo Clinic EHR system relies upon Epic for electronic management of health records which is used by providers across its campuses. For this analysis, we considered a database of 2.2 million clinical notes covering all of the unstructured text written, typed, or dictated into the Epic system during the course of clinical care, including but not limited to: progress notes, discharge summaries, inpatient notes, outpatient notes, and telephone call transcripts.
Data analysis	Statistical significance tests were run using the software package scipy v1.5.4 in Python. The software package sklearn v0.20.3 in Python was used to train logistic regression models for the propensity score matching analysis.

For manuscripts utilizing custom algorithms or software that are central to the research but not yet described in published literature, software must be made available to editors and reviewers. We strongly encourage code deposition in a community repository (e.g. GitHub). See the Nature Research guidelines for submitting code & software for further information.

Data

Policy information about availability of data

All manuscripts must include a data availability statement. This statement should provide the following information, where applicable:

- Accession codes, unique identifiers, or web links for publicly available datasets
- A list of figures that have associated raw data
- A description of any restrictions on data availability

Reasonable requests for de-identified data made to the corresponding author will be reviewed and processed by the Mayo Clinic institutional review board upon publication of this manuscript.

Field-specific reporting

Please select the one below that is the best fit for your research. If you are not sure, read the appropriate sections before making your selection.

☒ Life sciences ☐ Behavioural & social sciences ☐ Ecological, evolutionary & environmental sciences

For a reference copy of the document with all sections, see nature.com/documents/nr-reporting-summary-flat.pdf

Life sciences study design

All studies must disclose on these points even when the disclosure is negative.

Sample size	The sample size was determined based upon the number of patients in the Mayo Clinic EHR database at the time of the study in order to maximize the power of the downstream statistical tests.
Data exclusions	Patients who declined to give research authorization were excluded from the study. No patients were excluded on the basis of age, sex, race, ethnicity, or other clinical parameters.
Replication	No follow-up clinical experiments were performed to verify the findings in this study.
Randomization	Propensity score matching was performed in order to control for potential confounding factors when comparing the COVID-positive and COVID-negative cohorts.
Blinding	Blinding was not relevant due to the observational nature of this study.

Reporting for specific materials, systems and methods

We require information from authors about some types of materials, experimental systems and methods used in many studies. Here, indicate whether each material, system or method listed is relevant to your study. If you are not sure if a list item applies to your research, read the appropriate section before selecting a response.

Materials & experimental systems

n/a	Involved in the study	
☒	☐	Antibodies
☒	☐	Eukaryotic cell lines
☒	☐	Palaeontology and archaeology
☒	☐	Animals and other organisms
☐	☒	Human research participants
☒	☐	Clinical data
☒	☐	Dual use research of concern

Methods

n/a	Involved in the study	
☒	☐	ChIP-seq
☒	☐	Flow cytometry
☒	☐	MRI-based neuroimaging

Human research participants

Policy information about studies involving human research participants

Population characteristics	The population characteristics are described in Table 1.
Recruitment	This was an observational study of 1,803 hospitalized COVID-19 positive patients (positive PCR for SARS-CoV-2) in the Mayo Clinic electronic health record (EHR) database from March 12, 2020 to September 15, 2020. The sample size was determined based upon the number of patients in the Mayo Clinic EHR database at the time of the study in order to maximize the power of the downstream statistical tests. Patients who declined to give research authorization were excluded from the study. No patients were excluded on the basis of age, sex, ethnicity, or other clinical parameters.
Ethics oversight	Mayo Clinic Institutional Review Board

Note that full information on the approval of the study protocol must also be provided in the manuscript.
